# P2X7 Interactions and Signaling – Making Head or Tail of It

**DOI:** 10.3389/fnmol.2019.00183

**Published:** 2019-08-07

**Authors:** Robin Kopp, Anna Krautloher, Antonio Ramírez-Fernández, Annette Nicke

**Affiliations:** Walther Straub Institute of Pharmacology and Toxicology, Faculty of Medicine, LMU Munich, Munich, Germany

**Keywords:** C-terminus, protein-protein interaction (PPI), signaling/signaling pathways, P2X7 (purino) receptor, network

## Abstract

Extracellular adenine nucleotides play important roles in cell–cell communication and tissue homeostasis. High concentrations of extracellular ATP released by dying cells are sensed as a danger signal by the P2X7 receptor, a non-specific cation channel. Studies in P2X7 knockout mice and numerous disease models have demonstrated an important role of this receptor in inflammatory processes. P2X7 activation has been shown to induce a variety of cellular responses that are not usually associated with ion channel function, for example changes in the plasma membrane composition and morphology, ectodomain shedding, activation of lipases, kinases, and transcription factors, as well as cytokine release and apoptosis. In contrast to all other P2X family members, the P2X7 receptor contains a long intracellular C-terminus that constitutes 40% of the whole protein and is considered essential for most of these effects. So far, over 50 different proteins have been identified to physically interact with the P2X7 receptor. However, few of these interactions have been confirmed in independent studies and for the majority of these proteins, the interaction domains and the physiological consequences of the interactions are only poorly described. Also, while the structure of the P2X7 extracellular domain has recently been resolved, information about the organization and structure of its C-terminal tail remains elusive. After shortly describing the structure and assembly of the P2X7 receptor, this review gives an update of the identified or proposed interaction domains within the P2X7 C-terminus, describes signaling pathways in which this receptor has been involved, and provides an overlook of the identified interaction partners.

## Introduction

**FIGURE 1:**
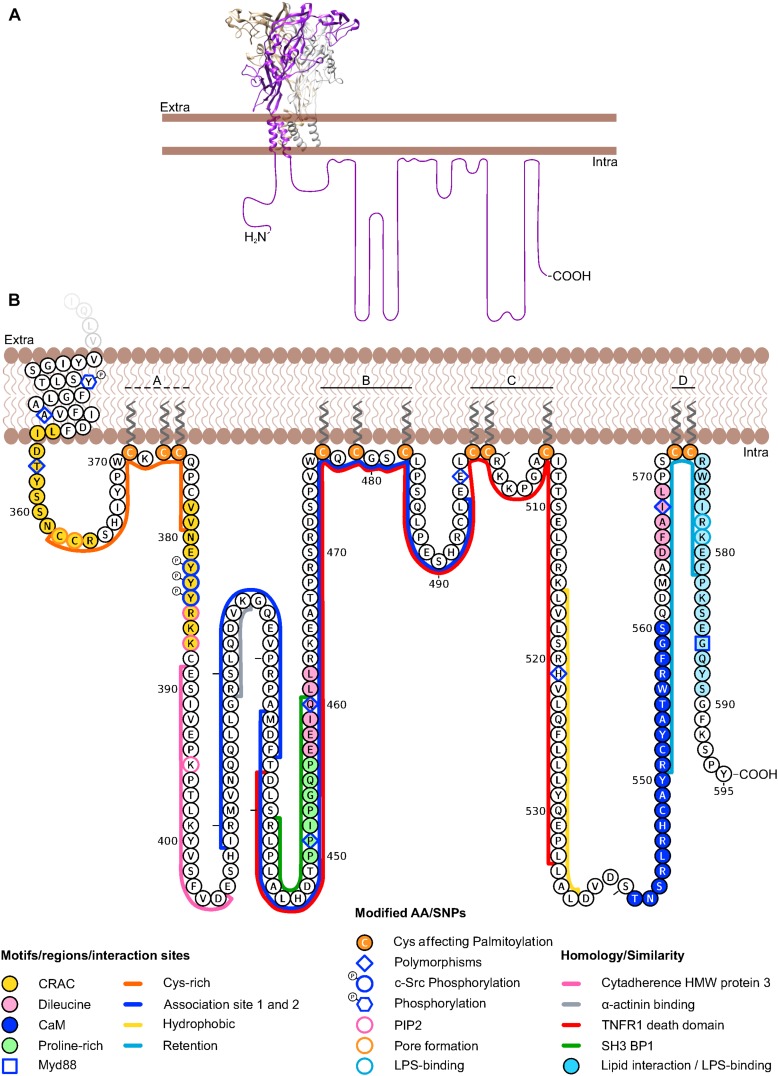
Schematic representation of the human P2X7 receptor depicting details of its intracellular C-terminus. **(A)** Homology model (https://swissmodel. expasy.org/) based on the crystal structure of the panda P2X7 in the closed, apo state (PDB ID: 5U1L) is shown with the three P2X7 subunits represented in gray, brown, and purple. Note the dolphin-shaped structure of a single subunit with the transmembrane regions representing the fluke. The structure of the intracellular C-terminus has not been determined. The estimated length of one unfolded C-terminus is represented in scale to the crystallized domains. **(B)** Amino acid sequence of the human P2X7 intracellular C-terminus representing individual amino acid residues and domains discussed in the text. Note that some of the motifs/domains are putative and species variations are not considered. Groups of palmitoylated cysteines (A–D) according are shown. A dashed line indicates partial palmitoylation (Gonnord et al., 2008). The aa sequence was drawn with Protter and manually modified: http://wlab.ethz.ch/protter/ (Omasits et al., 2014).

The ATP-gated P2X receptors are trimeric ion channels with inter-subunit ATP-binding sites (Kaczmarek-Hájek et al., 2012). In contrast to most other ion channel families, six of the seven cloned subunits can form functional homomeric receptors but heteromeric receptors have also been identified, for example P2X2/3 and P2X1/5 receptors (Saul et al., 2013). A single P2X subunit has been structurally compared to a dolphin (Figure 1A) and contains two α-helical transmembrane domains (fluke) that are linked by a large extracellular domain [269–288 amino acids (aa) long] that is mostly formed by β-sheets (body) and several loop domains (head, dorsal fin, and flippers) (Kawate et al., 2009). The intracellular N- and C- termini are less conserved than the rest of the protein. The N-termini are 20–45 aa long, while the intracellular C-termini are more variable in length with 29–87 aa for most subunits. P2X2, and in particular P2X7 contain considerably longer C-termini of 113/125 (human/mouse) and 240 aa, respectively. Human P2X5 and P2X6 have C-terminal sequences of 82 and 87 aa, respectively. Only in case of the ATP-bound open state of the human P2X3 receptor, a structure of these intracellular domains has been obtained so far (Mansoor et al., 2016). In this ATP-bound structure, the N- and C-termini form a network of three β-sheets (each formed by the C-terminus of one subunit and the N-termini of the two neighboring subunits) that is capping the cytoplasmic side of the pore. The termini appear to be disordered and flexible in the apo state.

The P2X7 receptor differs not only structurally but also functionally from all other P2X subtypes. In comparison, it has 10 to 100-fold reduced ATP sensitivity, suggesting that it functions as a “danger signal” detector for high ATP concentrations that are released at sites of tissue damage (Linden et al., 2019). A P2X7 splice variant (P2X7k) has been identified in rodents but not in humans, that can also be activated by extracellular nicotinamide adenine dinucleotide (NAD) via covalent enzymatic modification (ADP ribosylation) (Schwarz et al., 2012; Xu et al., 2012). The P2X7k variant also shows a higher ATP sensitivity.

Also, unlike other ion channels, P2X7 activation does not only open a non-selective cation channel, but in addition mediates a membrane permeability increase by forming a so-called “macropore” that can reach a diameter of 8.5 Å and allows the passage of large molecules such as the fluorescent dyes ethidium and YO-PRO1 (Di Virgilio et al., 2018b). P2X7 activation furthermore initiates a variety of signaling cascades that trigger caspase activation and cytokine release, plasma membrane reorganization, ectodomain shedding, and cell death to only name a few. Some of these effects are likely consequences of P2X7-dependent Ca^2+^ influx and/or K^+^ efflux, although a detailed description of the molecular interactions and signaling complexes involved is generally lacking. The C-terminus appears to be required for most of these effects and probably plays a role in positioning of the receptor in membrane microdomains (e.g., lipid rafts (Murrell-Lagnado, 2017) and/or signaling complexes (Kim et al., 2001)) that allow efficient signaling and/or direct interaction with signaling molecules.

It has to be mentioned, that considerable differences exist in the pharmacology of rat, mouse, and human P2X7 (Donnelly-Roberts et al., 2009). For example, at the rat isoform ATP and BzATP were 8 and 70 times more potent than at the mouse isoform and 10 and 25 times more potent than at the human isoform, respectively. While this could be attributed to single aa differences in the ligand-binding ectodomain of rat and mouse P2X7 (Young et al., 2007), a positive effect of the intracellular C-terminus on BzATP potency was shown in P2X7 chimeras, in which the human P2X7 C-terminus was replaced by the respective rat sequence (Rassendren et al., 1997). Likewise, sensitivity to divalent cations, dye-uptake efficiency and selectivity, and current kinetics largely differ between human and rodent isoforms and also between different cell types (Rassendren et al., 1997; Hibell et al., 2001; Janks et al., 2019) with the human and mouse isoforms being less efficient in dye uptake. However, despite these differences, the principal effects of P2X7 activation, such as dye uptake, interleukine-1β (IL-1β) release, phosphatidylserine-flip (PS)-flip, and blebbing appear to be present in all isoforms. Nevertheless, a systematic comparison is urgently needed.

P2X7 receptors are highly expressed in immune cells (in particular macrophages, T-cells, mast cells, and microglia), epithelial cells, oligodendrocytes of the CNS, and Schwann cells of the PNS (Di Virgilio et al., 2018a; Kaczmarek-Hájek et al., 2018). Their presence in neurons is more controversial (Illes et al., 2017; Miras-Portugal et al., 2017). Numerous studies describe P2X7 expression and function in astrocytes (Ballerini et al., 1996; Duan et al., 2003; Narcisse et al., 2005; Sperlagh et al., 2006; Norenberg et al., 2010; Oliveira et al., 2011; Sperlagh and Illes, 2014) while there is also contradictory evidence (Jabs et al., 2007). Thus, P2X7 detection in these cell types might depend on factors such as the species, tissue, model system, and activation state, or disease phase that is investigated. Furthermore, interpretation of findings depends on the sensitivity and/or specificity of the detection methods, as specificity of the most widely used antagonists (oxidized ATP and brilliant blue G) and the available P2X7 antibodies is questionable and proper control experiments (for example using P2X7 knockout animals) need to be performed (Sim et al., 2004; Anderson and Nedergaard, 2006).

The best-investigated and most widely accepted P2X7 functions are its roles in inflammation and immune signaling. Blockade or genetic ablation of the P2X7 receptor has early confirmed, that it is a major trigger of processing and release of pro-inflammatory IL-1β and resulted in amelioration of disease parameters in various experimental models ranging from inflammatory processes induced by infection, allograft rejection, and autoimmune diseases to numerous models of tissue or organ damage as well as various neurological diseases (Burnstock and Knight, 2018; Savio et al., 2018). In addition to its role in immune function and inflammation, which is often associated with the deleterious effects of its activation, P2X7 has also been shown to exert trophic roles, for example in microglia (Monif et al., 2009) or different cancer cells (Orioli et al., 2017). In humans, a truncated splice variant was identified that lacks the C-terminus and appears to serve mainly trophic functions (Adinolfi et al., 2010). In the following, we will provide an overview of the direct and indirect protein interactions and signaling pathways in which P2X7 has most commonly been involved.

## Interaction of the P2X7 Receptor With the P2X4 Receptor

Within the P2X receptor family, P2X4 shows the highest sequence similarity to P2X7 (47% amino acid identity of the human isoforms). The *P2rx4* gene is located just downstream of the *P2rx7* gene and they are thought to have originated from the same gene by gene duplication (Dubyak, 2007; Hou and Cao, 2016). Both subtypes show a widely overlapping expression pattern, in particular in immune cells and epithelial cells (Guo et al., 2007; Kaczmarek-Hájek et al., 2012), and have been linked to similar physiological and pathophysiological functions in inflammatory processes, such as reactive oxygen species (ROS) production and the secretion of mature IL-1β and IL-18 through the activation of the NLRP3 inflammasome (Babelova et al., 2009; Kawano et al., 2012; Hung et al., 2013). For example, P2X4 was shown to affect the P2X7-mediated maturation and release of IL-1β, (Pérez-Flores et al., 2015) and a rapid initial P2X4-mediated Ca^2+^ influx was suggested to initiate this cascade (Sakaki et al., 2013). Both receptors have also been involved in phagosome function (Qureshi et al., 2007; Kuehnel et al., 2009), autophagy, macrophage death (Kawano et al., 2012), as well as autocrine and paracrine activation of T cells via ATP-induced Ca^2+^ influx (Schenk et al., 2008; Yip et al., 2009; Woehrle et al., 2010; Manohar et al., 2012; Wang et al., 2014).

While heteromerisation of both subunits in trimeric complexes (Guo et al., 2007) was not confirmed (Torres et al., 1999; Nicke, 2008; Boumechache et al., 2009; Antonio et al., 2011), a number of studies provide evidence in favor of a direct physical association of both receptor types and/or a mutual functional interaction between both subtypes. Thus, both subunits could be co-immunoprecipitated from transfected cells, as well as various cell lines and primary cells (Guo et al., 2007; Boumechache et al., 2009; Weinhold et al., 2010; Hung et al., 2013; Pérez-Flores et al., 2015) and FRET studies on *Xenopus laevis* oocyte- and HEK293 cell-expressed subunits support a close association or heteromerisation (Pérez-Flores et al., 2015; Schneider et al., 2017). A close proximity within transfected HEK293 cells was also shown by *in situ* proximity ligation assays (Antonio et al., 2011). Functional evidence for an interaction was described in native and recombinant mammalian cells (Ma et al., 2006; Guo et al., 2007; Casas-Pruneda et al., 2009; Kawano et al., 2012; Pérez-Flores et al., 2015) but not in a more recent study (Schneider et al., 2017) in *Xenopus laevis* oocytes. Finally, a mutual interrelation between P2X4 and P2X7 mRNA and protein expression levels was described in kidney, E10 alveolar epithelial cells, and bone marrow derived dendritic cells (Weinhold et al., 2010; Craigie et al., 2013; Zech et al., 2016). To evaluate these results, it has to be considered, however, that the P2X4 subtype is mostly found intracellularly and co-localized with lysosomal markers (Bobanovic et al., 2002; Guo et al., 2007; Qureshi et al., 2007), whereas P2X7 is generally localized at the plasma membrane. Nonetheless, upon stimulation of the respective cells [e.g., via lipopolysacharide (LPS), CCL2/12 or ionomycin] an increased fraction of P2X4 receptors was found at the cell surface (Qureshi et al., 2007; Boumechache et al., 2009; Toulme et al., 2010; Toyomitsu et al., 2012).

## Structure of the P2X7 C-Terminus and Its Involvement in P2X7 Signaling

The P2X7 C-terminus constitutes about 40% of the whole P2X7 protein (Figure 1) and amino acid sequence identity between rat, mouse, and human C-termini is 80%. Except for the domains described below, the so-called P2X7-tail shows no sequence homology to other proteins. It is supposed to be localized intracellularly, but contains a lipophilic stretch of 21 aa (residues 516–536 in human P2X7) that would be long enough to form another transmembrane domain or reentry loop. Deletion or truncation of the majority of this intracellular tail prevents P2X7-mediated effects such as dye uptake (Surprenant et al., 1996) and plasma membrane blebbing (Wilson et al., 2002), and alters channel kinetics (Becker et al., 2008), but does not impair cell surface expression or ion channel function (Smart et al., 2003; Becker et al., 2008). In the following, we will shortly explain the current understanding of P2X7 pore formation and then describe identified domains and motifs within the P2X7 tail, starting from the very terminus toward the second TM domain.

### Pore Formation

A hallmark feature of P2X7 activation is the formation of a non-selective macropore. Both, naturally occurring splice variants of P2X7 and *in vitro* experiments with C-terminally truncated P2X7 receptors demonstrated that this property requires the C-terminus (Surprenant et al., 1996; Adinolfi et al., 2010). Basically two mechanisms of pore formation were discussed: According to the “pore dilation” hypothesis, pore formation is an intrinsic channel property and the consequence of a permeability increase from an initially cation selective channel to a non-selective pore. Alternatively, direct or indirect interaction with other pore forming proteins was suggested, with the large transmembrane channel Pannexin 1 (Panx1) representing the most prominent candidate (Pelegrin and Surprenant, 2006) (see below). Noteworthy, permeability to larger molecules like YO-PRO-1 or *N*-methyl-D-glucamine (NMDG) was also observed for P2X2 and P2X4 family members (Khakh et al., 1999; Virginio et al., 1999). However, at least for the P2X2 receptor this property appeared intrinsic to the receptor (Khakh and Egan, 2005; Chaumont and Khakh, 2008) and it was later shown that the time-dependent shift in the reversal potential of extracellular NMDG, that was generally interpreted as an increase in pore diameter, can also be the result of changes in intracellular ion concentration during whole-cell patch-clamp recordings (Li et al., 2015). While the mechanism of pore formation in P2X7 has been a long-standing debate (excellently reviewed in Di Virgilio et al., 2018b), more recent electrophysiological, photochemical, and biochemical experiments indicate that the pathway for larger molecules like NMDG or spermidine is also intrinsic to the P2X7 receptor and, similar to P2X2 (Li et al., 2015), the P2X7 channel is upon activation immediately permeable to both, small cations and large molecules (Riedel et al., 2007; Browne et al., 2013; Harkat et al., 2017; Karasawa et al., 2017; Pippel et al., 2017). Whether a P2X7-activated pathway for large anions that is observed in some cell types is also intrinsic to the P2X7 protein or mediated by a separate channel or pore, remains to be determined (Ugur and Ugur, 2019).

### Trafficking and Lipid Interaction Domains (∼Residues 540–595)

In the search for domains in the P2X7 C-terminus that control P2X7 channel function, pore forming properties, and plasma membrane expression, truncated P2X7 versions were investigated (Smart et al., 2003) and it was found that 95% (i.e., the sequence up to residue 581) of the rat P2X7 C-terminus are required to mediate ethidium uptake in HEK293 cells. Truncations between aa 551–581 as well as some single point mutations (C572G, R574G, or F581G) in this region resulted in a loss of surface expression. Upon further truncation (residues 380–550), the ion channel but not the pore activity was regained. Thus it was suggested, that amino acid residues 551–581 contain a *retention motif* that is generally masked but becomes exposed by truncations or single point mutations in this region and then inhibits surface expression. In support of a role of this region in receptor trafficking, the loss-of-function polymorphism I568N in this region of the human P2X7 was also found to inhibit cell surface expression (Wiley et al., 2003).

The supposed retention/retrieval region overlaps with a *lipid interaction or putative LPS-binding motif* (residues 574–589) that is homologous to the LPS binding domains of LPS-binding protein (LBP 44% identity) and bactericidal permeability-increasing protein (BPI 31% identity) and was shown to bind LPS *in vitro* (Denlinger et al., 2001). Both surface expression and LPS binding were abolished when the basic residues R578 and K579 were mutated in human P2X7 (Denlinger et al., 2003). LBP and BPI are pattern recognition proteins that, upon LPS-binding, can stimulate a defensive host response to gram-negative bacteria, although in different ways: LBP is a plasma protein, that increases the host cell’s sensitivity to endotoxins by disaggregating, transporting, and binding LPS to other LPS-binding proteins, such as the pattern recognition receptor CD14. CD14 is a glycosylphosphatidylinositol (GPI)-anchored receptor that acts as a co-receptor for the toll-like receptor (TLR) 4 complex (Ranoa et al., 2013). Upon LPS binding, TLR4 induces via the adaptor protein myeloid differentiation primary-response protein 88 (MyD88) and the transcription factor NF-κB cytokine production. Interestingly, the very C-terminus of mouse P2X7, in particular G586 was described to directly interact with MyD88 (Liu et al., 2011) (see also Section “Proteins Involved in P2X7-Mediated Interleukin Secretion”).

The soluble BPI has anti-endotoxin and direct bactericidal properties against gram-negative bacteria and can neutralize LPS, thereby inhibiting LPS-triggered cytokine production and an overshooting immune response (Weiss, 2003). Cytosolic LPS, experimentally delivered by cholera toxin B or by transfection of mouse bone marrow-derived macrophages, was shown to decrease the threshold for ATP-induced P2X7-associated pore opening, supposedly by allosteric modulation via the putative LPS binding motif in the P2X7 C-terminus (Yang et al., 2015). Internalization of LPS is facilitated by CD14. Accordingly, the presence of CD14 resulted in an increased co-localization of LPS and P2X7 in transfected HEK293 cells. A direct interaction between P2X7 and CD14 was also reported (Dagvadorj et al., 2015) (see also Section “Proteins Involved in P2X7-Mediated Interleukin Secretion”).

Signaling requires the spatial organization (co-localization or sequestration) of its components in subcellular environments for example by protein scaffolds or membrane domains. The lipid interaction motif in P2X7 was not only suggested to serve as a binding domain for LPS, but also for phospholipids and thereby modulating the receptor’s localization (Denlinger et al., 2001), for example in lipid rafts. An association between P2X7 and lipid rafts was found in T-cells, where P2X7 is ADP-ribosylated by ART2.2 (Bannas et al., 2005), in mouse lung alveolar epithelial cells (Barth et al., 2007), and in rat submandibular gland cells. In the latter, a lipid-raft pool and a non-raft fraction of P2X7 receptors were described that couple to different signaling pathways (Garcia-Marcos et al., 2006a). This would be in accordance with studies, showing that P2X7 modulates phospholipase A2, C, and D (el-Moatassim and Dubyak, 1992; Hung and Sun, 2002; Garcia-Marcos et al., 2006b; El Ouaaliti et al., 2012) (see also Section “P2X7 – Mediated Lipase Activation and Lipid Interactions”). Also functional regulation of P2X7 by phosphatidylinositol 4,5-bisphosphate (PIP2) was shown in patch-clamp experiments with *Xenopus laevis* oocytes. Although no direct binding of the P2X7 C-terminus and PIP2 could be observed, residues R385, K387 and K395 of the human P2X7 receptor were shown to be important for the interaction with PIP2 (Zhao et al., 2007).

Just upstream of the LPS binding motif, Gonnord et al. (2008) identified two *palmitoylated cysteine residues* (C572, C573) in mouse P2X7 that were essential for P2X7 surface trafficking. Likewise, two more proximal groups of cysteine residues (C477, C479, C482 and C498, C499, C506) were palmitoylated and required for surface expression. Mutation of the juxtamembrane cysteine residues C371, C373, and C374, however, showed only a 50% decrease in palmitoylation and reduced surface localization (Gonnord et al., 2008). Palmitoylation is a reversible posttranslational modification that increases membrane association and can also affect function, stability, and subcellular trafficking of proteins into membrane compartments, as for example the cholesterol- and sphingolipid-enriched lipid rafts.

Interestingly, the permeability of the P2X7 appears to depend not solely on the C-terminus, but also on the lipid composition of the cell membrane (Karasawa et al., 2017). In *in vitro* experiments with purified truncated panda P2X7 in artificial liposomes, phosphatidylglycerol and sphingomyelin facilitated YO-PRO-1 permeation, whereas cholesterol had an inhibitory effect. It was concluded that the palmitoylated cysteine residues in full-length receptors prevent the inhibitory effect of cholesterol by shielding the TM domains, while in C-terminally truncated P2X7, cholesterol can interact with the transmembrane helices and thereby limits its permeability. Thus, the pore forming properties of the P2X7 could be influenced by modulation of the membrane composition and may be cell-type specific (Di Virgilio et al., 2018b).

An unusual Ca^2+^-dependent *calmodulin (CaM) binding motif* was functionally and biochemically identified in HEK293 cell-expressed rat P2X7 [residues I541-S560, Roger et al., 2008). In this sequence ([I-x(3)-L-x(10)-W]), key bulky amino acid residues form a 1-5-16 motif. CaM is a calcium sensor that modulates the function of a wide variety of enzymes and ion channels, but can also act as an adaptor, interacting with other target proteins (Villalobo et al., 2018). CaM binding to P2X7 was found to facilitate currents and blebbing. In human P2X7, both the CaM binding motif and current facilitation were not detected, but could be reconstituted by replacement of critical residues (T541I, C552S, and G559V) (Roger et al., 2010). P2X7 signaling via Ca^2+^calmodulin-dependent kinase II (CaMKII) was also shown (Diaz-Hernandez et al., 2008; Gomez-Villafuertes et al., 2009).

### The Death Domain (∼Residues 430–530)

Based on comparative sequence analysis, residues 438–533 of the human P2X7 were found to be similar (20% identity, 50% conservation) to the *death domain (DD)* of the human tumor necrosis factor receptor 1 (TNFR1) (Denlinger et al., 2001). The DD is a subclass of protein motifs known as the death fold. It is a protein interaction domain that is contained in numerous proteins and enables them to oligomerize. Many DD-containing proteins are involved in apoptosis and inflammation.

Within the postulated P2X7 DD homology domain, a *proline-rich region* (residues 450–456) contains two overlapping PxxP motifs and may represent a canonical binding site for *cellular sarcoma tyrosine kinase (c-Src) homology 3 (SH3) domains* (Watters et al., 2001). An alignment (ClustalW) between the human SH3-domain binding protein 1 (Q9Y3L3) and residues 441–460 of the human P2X7 receptor shows a 40% sequence identity, in agreement with (Denlinger et al., 2001). SH3 domains are approximately 60 aa long modules that mediate protein interactions and are involved in various intracellular signaling pathways. They recognize *proline-rich regions* containing the PxxP motif (Kurochkina and Guha, 2013) and are present in phospholipases, tyrosine kinases and other signaling proteins (Kaneko et al., 2008). The interaction between the scaffolding protein MAGuK and the P2X7 receptor was suggested to be mediated via SH3 domains, but evidence is lacking (Kim et al., 2001).

There are also two sequences (457–462 and 565–569) with similarities to *a dileucine motif* ([D/E]xxxL[I/L]) (Wiley et al., 2011). This short signaling motif allows for interaction between cargo proteins and adaptor proteins for trafficking and controls endosomal sorting (Kozik et al., 2010).

C-terminal truncation of the human P2X7 at positions 408, 436, and 505 (Becker et al., 2008) lead to reduced ATP-induced inward currents and loss of its biphasic activation and deactivation kinetics when expressed in *Xenopus laevis* oocytes. In case of the 1–436 and 1–505 core receptors, the electrophysiological phenotype of the full-length receptor could be reconstituted by co-expression of a soluble tail construct (residues 434–595). Based on affinity purification, BN-PAGE, and cross-linking experiments a stable *association between the regions* 409–436 and 434–494 was identified, which provides the first information on molecular interactions within the P2X7 tail.

Between the sequence with homology to the death domain and the juxtamembrane region (see below) two regions with homology to *binding sites for cytoskeletal proteins* have been identified. Residues 389–405 of human P2X7 show 53% identity with the *cytadherence high molecular weight protein 3* from *Mycoplasma genitalium*, which binds actin filaments (Denlinger et al., 2001; Watters et al., 2001). Residues 419-425 in rat P2X7 (KSLQDVK) are homologous to the *α-actinin 2 binding sequence in the glutamate receptor NR1 subunit.* In support of a close interaction with the cytoskeleton, the cytoskeletal proteins α-actinin 4 and supervillin, which both interact directly with β-actin were identified in a search for possible interaction partners of rat P2X7 (Kim et al., 2001; Gu et al., 2009).

### Juxtamembrane Region(s) (Residues 1–26 and 357–387)

Deletion of the juxtamembrane *cysteine-rich domain* (residues 362–379) was shown to affect the permeation and/or pore forming properties of rat and human P2X7 (Jiang et al., 2005; Robinson et al., 2014). Additionally, it was shown in a chimeric approach (Allsopp and Evans, 2015), that both the N- and the C-terminal juxtamembrane regions of the human P2X7 (including the cysteine-rich domain) are important for pore formation and regulation of channel kinetics. In the panda P2X7, serine-substitutions of C362 and C363 in this region resulted in complete inhibition of YO-PRO-1 uptake (Karasawa et al., 2017). Upstream (residues 354-364) and downstream (residues 378-387) of this cysteine-rich domain, there are at least two *cholesterol recognition amino acid consensus (CRAC) motifs* [(L/V)X1–5 YX1–5(K/R)] that are conserved in human and rodent P2X7. Further CRAC motifs have been identified in the N-terminus, the extracellular end of TM1, and the C-terminus of P2X7 (Robinson et al., 2014). Based on these findings, it was suggested that the juxtamembrane cysteine-rich domain framed by the CRAC motifs could alter the tilting angle of TM2 or act as a membrane anchor and thereby facilitate movements required for channel and/or pore opening (Allsopp and Evans, 2015; Karasawa et al., 2017). A similar anchor-like function that stabilizes the open state was ascribed to the cytoplasmic cap in the human P2X3, which is assumed to undergo profound reorganization upon channel activation (Mansoor et al., 2016).

Within the distal CRAC motif, three neighboring tyrosine residues (Y382-Y384) were identified that can be phosphorylated by the *c-Src tyrosine kinase* (Leduc-Pessah et al., 2017). In this study, it was found that Src kinase activation by morphine (via μ-opioid receptors) and subsequent P2X7 phosphorylation resulted in increased receptor expression and activity in rat spinal microglia. The resulting loss of morphine-induced analgesia linked P2X7 activity to the development of morphine tolerance.

### Single Nucleotide Polymorphisms (SNPs) in the P2X7-Tail

The human P2X7 is highly polymorphic (Bartlett et al., 2014). The *T357S* polymorphism was found by ATP-induced influx measurements to cause a partial loss of function in human monocytes, lymphocytes, and macrophages, and was associated with impaired mycobacterial killing (Shemon et al., 2006; Miller et al., 2011). Interestingly, this SNP resulted in a complete loss of function when occurring in homozygote constellation or in combination with another loss-of-function SNP. A loss-of function phenotype was confirmed in *Xenopus laevis* oocytes and HEK293 cells overexpressing the mutant T357S P2X7 (Shemon et al., 2006).

Based on genetic studies, the *human Q460R* polymorphism has been associated with bipolar disorders and major depressive disorder (Barden et al., 2006; Lucae et al., 2006; McQuillin et al., 2009). ATP-induced ethidium uptake measurements in Q460R P2X7-transfected HEK293 cells revealed a slight reduction in pore formation (Fuller et al., 2009; Stokes et al., 2010). Interestingly, careful functional studies showed that this SNP is not *per se* compromised in its function, but shows impaired Ca^2+^ influx, channel currents, intracellular signaling, and also affected the sleep quality in a humanized Q460R P2X7 knock-in mouse model, if co-expressed with the respective non-polymorphic variant (Aprile-Garcia et al., 2016; Metzger et al., 2017).

The *E496A* SNP was found to prevent ATP-induced ethidium uptake, Ba^2+^ permeation, and induction of apoptosis in human B lymphocytes and was associated with cancer metastasis (Gu et al., 2001; Ghiringhelli et al., 2009). When expressed in *Xenopus laevis* oocytes or HEK293 cells and analyzed electrophysiologically, however, the E496A substitution had no effect on the ion channel functions of the receptor (Boldt et al., 2013).

The human loss-of-function SNP, *I568N*, was reported to prevent receptor trafficking and cell surface expression [see also Section “Trafficking and Lipid Interaction Domains (∼Residues 540–595)”] (Wiley et al., 2003), supposedly because of its localization within a sequence [DFAI(568)L] (Wiley et al., 2011) similar to a dileucine motif – [D/E]xxxL[I/L]– [(Kozik et al., 2010), compare Section “The Death Domain (∼Residues 430–530)”].

A gain of function in pore formation and IL- 1β secretion has been reported for the *human A348T* SNP (Stokes et al., 2010), whereas *H521Q* has been reported to represent a neutral SNP (Wiley et al., 2011).

The *murine P451L* loss-of-function SNP was identified by comparison of T-cells from different mouse strains (Adriouch et al., 2002). This SNP is found in the commonly used C57BL/6 strain, but not in BALB/c mice, rats, or humans. It impairs ATP-induced cation fluxes, pore formation, PS externalization, NAD-sensitivity as well as lysis and apoptosis of thymocytes (Schwarz et al., 2012; Rissiek et al., 2015), and has been associated with reduced pain sensitivity (Sorge et al., 2012). It lies within the SH3-binding domain [compare Section “The Death Domain (∼Residues 430–530)”].

## P2X7 Mediated Signaling Pathways

A multitude of downstream events have been identified upon P2X7 activation. In the following, we will focus on the proteins involved in the P2X7 activated signaling pathways rather than the physiological consequences or cell types in which these have been observed.

### Release of IL-1β and Other Cytokines

The most investigated P2X7 function is probably its role in NLRP3 inflammasome assembly and subsequent maturation and release of IL-1β by macrophages and other immune cells. The pro-inflammatory IL-1β is a member of the interleukine-1 cytokine family, which comprises the IL-1 (IL-1α, IL-1β, IL-33, IL-1Ra), IL-18 (IL-18, IL-37), and IL-36 (IL-36Rα, IL-36α,β,γ, IL-38) subfamilies and includes pro- and anti-inflammatory cytokines (Dinarello, 2018). Due to its earlier identification and major role in host defense of the innate immune system and autoinflammatory diseases, IL-1β is so far best studied. Pro-IL-1β synthesis (and also that of NLRP3, see below) is induced by the transcription factor NF-κB, which in turn is activated upon binding of pathogen-associated molecular patterns (PAMPs), such as LPS, to the TLR4 (priming). Processing and release of mature IL-1β is then induced in a second step (activation) by inflammasome assembly and activation of caspase 1 by a diverse range of damage- or danger-associated molecular patterns (DAMPs), including ATP (Mariathasan et al., 2006).

Activation of caspase 1 by proteolytic conversion of pro-caspase 1 requires the NLRP3 inflammasome, a multiprotein complex that consists of the pattern recognition receptor NLRP3, the adaptor apoptosis-associated speck-like protein containing a caspase-recruitment domain (ASC), and the cysteine protease caspase 1 (Gross et al., 2011). In addition, never-in-mitosis A (NIMA)-related kinase 7 (NEK7) was recently identified as an essential component (He et al., 2016b; Shi et al., 2016).

K^+^ efflux and depletion was early shown to constitute a critical step in ATP-induced IL-1β production (Perregaux and Gabel, 1998) and generation of the first P2X7 knockout mouse clearly demonstrated the involvement of the P2X7 in this process (Solle et al., 2001). More recently, K^+^ depletion has been confirmed to represent an essential and sufficient requirement for inflammasome assembly induced by a diverse variety of DAMPs (Munoz-Planillo et al., 2013). However, while the P2X7 was generally assumed to represent the K^+^ conduit, a recent study identified the two-pore domain K^+^ channel TWIK2 as an ATP-responsive K^+^ efflux channel (Di et al., 2018). According to this study, P2X7-induced cation influx generates the driving force for K^+^ efflux. The molecular mechanisms of inflammasome assembly and caspase activation are little understood. Based on immunoprecipitation and co-localization studies in cell lines and primary mouse microglia, it has been suggested that the P2X7 is directly interacting with NLRP3 (Franceschini et al., 2015). Likewise, an interaction between P2X7 and the NLRP2 inflammasome was proposed in human astrocytes (Minkiewicz et al., 2013) (see also Section “Proteins Involved in P2X7-Mediated Interleukin Secretion”).

In addition to K^+^ depletion, cytosolic ROS production, either by NADPH oxidase or due to mitochondrial dysfunction, has been implicated in NLRP3 inflammasome activation and its exact role remains to be determined (He et al., 2016a). With the exception of the interleukine receptor antagonist (IL-1Ra), all IL-1 family members lack a signal peptide and are formed as precursor in the cytoplasm. Various mechanisms of non-classical IL-1β release mechanism including exocytosis via lysosomes, microvesicle shedding, exosome release, and release upon pyroptotic cell death have been proposed (for references and details see Dubyak, 2012; Giuliani et al., 2017). While PLC, PLA2 (Andrei et al., 2004), src kinase, p38, acid sphingomyelinase (Bianco et al., 2009), caspase 1 (Keller et al., 2008), and gasdermin (Evavold et al., 2018) have been involved, the exact mechanism(s) and P2X7 involvement remain(s) incompletely understood. In addition to IL-1β, numerous other cytokines, chemokines, and proteins have been shown to be released upon P2X7 activation (e.g., de Torre-Minguela et al., 2016).

### ROS Formation/Mitochondrial Function

ROS are continuously generated by the mitochondrial electron transport chain or by activation of NADPH oxidases (NOXs). They represent important signaling molecules under physiological conditions. Under pathological conditions, increased ROS production contributes to immune signaling and killing of phagocytosed microorganisms, but also to deleterious effects such as protein, lipid, and DNA modification and damage. Seven NOX family members are known and the respective NADPH oxidase complexes are subtype-specifically localized in internal and plasma membranes. They consist of the membrane integrated catalytic subunit with or without the p22phox protein and regulating cytosolic proteins including the Rho-GTPase Rac. NOX are activated by numerous receptors and NOX complex assembly and activity can be further regulated by Ca^2+^ signaling and subunit phosphorylation for example by protein kinase C isoforms, p38 and ERK1/2 MAP kinases, and phosphoinositide-3 kinase (PI3Ks) (Guerra et al., 2007; Spooner and Yilmaz, 2011; Haslund-Vinding et al., 2017; Belambri et al., 2018). P2X7-mediated NOX subunit phosphorylation and ROS production has been shown in microglia and macrophages (Parvathenani et al., 2003; Moore and MacKenzie, 2009; Lenertz et al., 2009) and few other cell types (Wang and Sluyter, 2013). The molecular mechanisms were suggested to involve kinase activation via Ca^2+^ influx (Guerra et al., 2007; Noguchi et al., 2008; Martel-Gallegos et al., 2013).

Interestingly, tonic stimulation by low levels of ATP was found to hyperpolarize the mitochondrial potential, increase mitochondrial Ca^2+^ content, and increase the cells’ ATP content in transfected cells. This effect was dependent on the C-terminus and proposed to be due to a P2X7-mediated constant but low level Ca^2+^ transfer into the mitochondria that stimulates trophic effects whereas strong P2X7 stimulation causes mitochondrial Ca^2+^ overload and collapse and results in cell death (Adinolfi et al., 2005). P2X7-expressing cells also upregulated the glucose transporter and glycolytic enzymes, showed increased glycolysis, oxidative phosphorylation, and protein kinase B phosphorylation, and were able to proliferate even in the absence of serum and glucose (Amoroso et al., 2012) (see also Di Virgilio et al., 2017).

### P2X7 – Mediated Lipase Activation and Lipid Interactions

Phospholipids, glycolipids, and cholesterol represent the major lipid components of animal plasma membranes. Cholesterol is an important constituent of lipid rafts and phospholipids can be broken down by phospholipases to produce different lipid second messengers or bioactive mediators of cellular signaling. Cholesterol as well as several phospholipases have been proposed to be involved in P2X7 signaling and function.

#### Phospholipase A2 (PLA2)

PLA2 phospholipases cleave phospholipids preferentially in the middle position of glycerol to release fatty acids and lysophospholipids.

Out of the six diverse groups of mammalian PLA2 enzyme families, the cytosolic PLA2α is the best-investigated enzyme. It belongs together with the β,γ,δ,ε, and ζ subtypes to the group IV family of cytosolic PLA2 (Leslie, 2015). cPLA2α preferentially catalyzes the hydrolysis of phospholipids to arachidonic acid and lysophospholipids, which are precursors for numerous bioactive lipids such as prostaglandins, leukotrienes, and epoxyeicosatrienoic acids (EETs). Ca^2+^-independent PLA2 (iPLA2, group VI) are similar to cPLA2 but do not require Ca^2+^ for activation. Both types are also implicated in the regulation of intracellular membrane trafficking by the induction of changes in the membrane curvature that is required for membrane budding (Leslie, 2015).

cPLA2α is widely expressed in all tissues and regulated by its transcriptional level (e.g., induced by Ras and MAPK pathways and NF-κB, hypoxia-inducible factor, Sp1, and c-Jun), Ca^2+^, and phosphorylation by MAPK. Ca^2+^ increase promotes its translocation to intracellular membranes, a requirement for arachidonic acid release. Phosphorylation by MAPKs can enhance its activity (Leslie, 2015). P2X7-mediated activation of cPLA2 and iPLA2 has been reported in immune and epithelial cells (Alzola et al., 1998; Chaib et al., 2000; Andrei et al., 2004; Kahlenberg and Dubyak, 2004; Garcia-Marcos et al., 2006b; Costa-Junior et al., 2011) and has been associated with various downstream effects such as PLD activation, kallikrein secretion, bioactive lipid generation, and pore formation as well as IL-1β processing, blebbing, and PS-flip (Garcia-Marcos et al., 2006b; Anrather et al., 2011; Costa-Junior et al., 2011; Norris et al., 2014; Wan et al., 2014; Alarcon-Vila et al., 2019; Janks et al., 2019). Janks et al. (2019) recently reported an involvement of undefined chloride channels downstream of PLA2 in some of these processes. The mechanism of PLA2 activation by P2X7 remains unclear but was suggested to involve MAP kinases, P-I4 kinase/PIP2 (Garcia-Marcos et al., 2006b; Wan et al., 2014) and/or n-SMase activation in lipid rafts (Garcia-Marcos et al., 2006a). In case of cPLA2, it might also be activated by Ca^2+^ influx through P2X7.

#### Phospholipase C (PLC)

In animals, PLC cleaves phosphatidylinositol-4,5-bisphosphate (PIP2) into the second messengers diacylglycerol and inositol-1,4,5,-triphosphate (Suh et al., 2008; Fukami et al., 2010). Besides PKC activation and mobilization of intracellular Ca^2+^ (via DAG and IP3, respectively) this process also influences the local concentration of PIP2 (an important membrane anchor and modulator of multiple processes and receptors) and the synthesis of the signaling molecule PIP3, which is generated by phosphatidylinositol 3-kinase (PI3K) from PIP2. Thirteen mammalian PLC isoforms that are organized in six groups are known and expressed in a tissue and/or cell-specific manner. In addition to the common catalytic and Ca^2+^-binding domains all but the PLCζ isotype contain pleckstrin homology (PH) domains that can mediate interactions with phosphatidylinositol lipids, G protein βγ subunits, or other proteins. Furthermore, some isotypes have specific domains that contribute to their individual functions: thus the src homlogy (SH) domains in PLCγ allow its interaction with and activation by receptor and cytosolic tyrosine kinases. Ras-associating domains and Ras-GTPase exchange factor-like domains in PLCε mediate its interactions with members of the Ras family of small G proteins, and the long C-terminus of PLCβ contains determinants for Gq protein interactions, membrane binding, and nuclear localization (Suh et al., 2008; Fukami et al., 2010).

Several GPCRs, including some P2Y receptors, activate PLC. However, few reports exist on the activation of PLC by P2X7 (Carrasquero et al., 2010) and K^+^ depletion has been suggested as a mechanism (Andrei et al., 2004; Clark et al., 2010). Also, modulation of PLC downstream effects by P2X7 has been reported but appears to be indirect and not dependent on influx of extracellular Ca^2+^ (Garcia-Marcos et al., 2006b). In microglia, for example, it was found that P2X7-induced Ca^2+^ rise increases DAG lipase activity and thus favors production of the endocannabinoid 2-AG from DAG, which is generated by PLC (Witting et al., 2004). A negative modulation of P2X7 through the depletion of PIP2 (supposedly due to PLC) has also been reported (Zhao et al., 2007) and three residues in the C-terminus (R385, K387, K395) might be involved in this interaction. However, the mechanism could also be indirect as no direct PIP2-P2X7 interaction was identified (Bernier et al., 2013) [compare Section “Trafficking and Lipid Interaction Domains (∼Residues 540–595)”].

#### Phospholipase D (PLD)

PLDs represent a family of phosphodiesterases that catalyze the removal of head groups from glycerophospholipids (typically phosphatidylcholine), thereby generating the regulatory molecule phosphatidic acid (PA). More generally, this process represents a headgroup exchange by water and in the presence of primary alcohols generates phosphatidylalcohol. PA, due to its small negatively charged headgroup, can induce negative curvature of membranes if sufficient concentrations are reached. In addition, PAs can act as lipid anchors for numerous PA binding proteins and can modulate/activate various proteins, such as the NOX complex, kinases, PLC and G-protein regulatory proteins, to only name a few (Bruntz et al., 2014). PA can also be converted to DAG and lysophosphatidic acid.

In mammals, the two isoforms PLD1 and PLD2 occur almost ubiquitously, associate with membranes, and participate in processes that involve membrane remodeling such as vesicular transport and endocytosis but also many others. PLDs are activated by a variety of receptors (GPCRs, receptor tyrosine kinases, and integrins) and signaling molecules. Direct interaction and activation has been shown for PKC and the small Ras GTPases RhoA and ARF (Selvy et al., 2011; Bruntz et al., 2014). In a macrophage cell line, P2X7 activation was found to induce rapid PLD activation that was only partially dependent on Ca^2+^ and PKC (Humphreys and Dubyak, 1996) and subsequent studies in human and mouse macrophages showed that P2X7-dependent killing of intracellular pathogens requires PLD activation (Kusner and Adams, 2000; Fairbairn et al., 2001; Coutinho-Silva et al., 2003). In thymocytes, Ca^2+^-dependent activation of PLD by P2X7 was shown (Le Stunff et al., 2004). What links P2X7 to PLD activation is not known in detail but influx of bivalent cations (Gargett et al., 1996), kinases (Hung and Sun, 2002; Perez-Andres et al., 2002; Pochet et al., 2003), and small G-protein interactions via the putative SH3 domain (Denlinger et al., 2001) have been involved.

#### Sphingomyelinase

Sphingomyelin is a phospholipid based on the unsaturated aminoalcohol sphingosine instead of glycerol. It is the most abundant sphingolipid with particularly high levels in the CNS and constitutes a major component of the plasma membrane. Due to its ability to bind cholesterol, it plays an important role in the formation of lipid rafts. Its content in the cell is regulated by *de novo* synthesis in the ER/Golgi (a multistep process involving sphingomyelin synthases) and its degradation by sphingomyelinases (SMases). SMases hydrolize sphingomyelin to phosphocholine and ceramide (sphingosine coupled via an amide bound to a fatty acid), a bioactive molecule that is involved in apoptosis, cell cycle, organization of membrane domains (”ceramide platforms”), inflammation, and various diseases (Gomez-Munoz et al., 2016). In addition, ceramide can be metabolized to further bioactive sphingolipids, such as the mitogenic sphingosine-1-phosphate.

Six types of SMases have been identified and were grouped according to the optimal pH value for their activation into acidic, alkaline and four neutral SMases. Of these, the lysosomal acidic a-SMase and Mg^2+^-dependent neutral n-SMase2 are best characterized and considered the major candidates for ceramide production. n-SMase is located in Golgi and plasma membrane domains and regulated by transcription, anionic phospholipids, phosphorylation, and in response to several cytokines, including TNF-α and IL-1β (Shamseddine et al., 2015).

In thymocytes and macrophages, P2X7 has been involved in the *de novo* synthesis of ceramide and subsequent apoptosis (Lepine et al., 2006; Raymond and Le Stunff, 2006) and it was speculated that the P2X7 death domain might be involved in ceramide production in macrophages. Similarly, this domain was suggested to be involved in P2X7-induced activation of n-SMase in lipid rafts and subsequent PLA2 activation in submandibular gland cells. In a more recent study on astrocytes it was concluded that P2X7, via src kinase (maybe by interacting with the SH2 domain) and p38MAPK activation, induces translocation of a-SMase to the outer plasma membrane leaflet where it induces blebbing and shedding of IL-1β-containing micro particles (Bianco et al., 2009). It was also suggested that P2X7, via a-SMase activation can induce the rapid release of HIV-1-containing compartments from HIV-infected macrophages (Graziano et al., 2015).

### P2X7 Effects on Membrane Organization and Morphology

#### Phosphatidylserine Exposure (PS-Flip) and Shedding

In healthy cells, PS is distributed to the inner leaflet of the plasma membrane. So-called flippases, most likely P4-ATPase ATP11C and its chaperone CDC50A, are required to keep this asymmetry (Segawa et al., 2014). Under certain conditions, for example during apoptosis, PS is translocated to the cell surface by scramblases (Segawa and Nagata, 2015). Anoctamin-6/TMEM16F (Ano6) and Xk-related protein 8 (Xkr8) were identified as scramblases (Suzuki et al., 2010, 2013) and proposed to account for Ca^2+^-induced PS scrambling and a caspase/apoptosis-induced scrambling respectively (Suzuki et al., 2013). For the latter, simultaneous inactivation of ATP11C and activation of Xkr8 by caspases is required (Suzuki et al., 2013; Segawa et al., 2014).

Brief activation of P2X7 was shown to result in a reversible PS translocation, while prolonged activation results in irreversible exposure of PS and subsequent cell death (Mackenzie et al., 2005). A functional and physical interaction between P2X7 and the Ca^2+^ activated Cl^–^ channel Ano6 was identified and suggested to mediate the translocation of PS (Ousingsawat et al., 2015). However, the molecular mechanisms of this interaction are unclear and interaction between Ano6 and P2X7 was not confirmed in another study (Stolz et al., 2015).

Reversible PS flip is also part of a signal transduction pathway in response to pathological conditions and P2X7-mediated PS flip can lead to shedding of L-selectin (CD62L) (Elliott et al., 2005), a cell adhesion molecule that initiates leukocyte tethering, the first step of the adherens and migration cascade (Ivetic, 2018). Shedding of CD62L from human monocytes occurs precisely during transmigration and is important for the invasion and direction of migration (Rzeniewicz et al., 2015) and PS exposure increased the adhesion of cells to the endothelial cell layer (Manodori et al., 2000). Thus PS translocation appears to be relevant for leukocyte migration and P2X7-mediated PS flip might increase the membrane fluidity and plasticity of the cell and thereby facilitate the transmigration processes (Elliott et al., 2005; Qu and Dubyak, 2009).

In addition to CD62L (Jamieson et al., 1996; Gu et al., 1998; Labasi et al., 2002; Elliott et al., 2005; Sengstake et al., 2006; Scheuplein et al., 2009; Schwarz et al., 2012), shedding of low affinity immunoglobulin epsilon Fc receptor (CD23) (Gu et al., 1998; Chen et al., 1999; Sluyter and Wiley, 2002; Pupovac et al., 2015), complement receptor type 2 (CD21) (Sengstake et al., 2006), tumor necrosis factor receptor superfamily member 7 (CD27) (Moon et al., 2006), IL-6R (Garbers et al., 2011), CXCL16 (Pupovac et al., 2013), and vascular cell adhesion molecule 1 (VCAM-1) (Mishra et al., 2016) was reported upon P2X7 activation and was mainly linked to activation of membrane-associated metalloproteases, in particular the a disintegrin and metalloprotease domain-containing proteins (ADAM) 10 and ADAM17. Out of the 21 ADAM family members, these two have been studied the most. They are widely expressed by immune cells and their activity is controlled by multiple regulatory mechanisms (Grötzinger et al., 2017; Lambrecht et al., 2018). Interestingly, it was shown that PS exposure is required for ADAM17 activity (Sommer et al., 2016) and phosphorylation by ERK and p38 is important for its activation (Diaz-Rodriguez et al., 2002) and membrane trafficking (Soond, 2005), thus providing a direct link between metalloprotease activity and these described P2X7 signaling pathways.

#### Plasma Membrane Blebbing

Blebbing is the formation of spherical protrusions of the plasma membrane. It requires the detachment and/or local rupture of the actomyosin cortex from the membrane (bleb nucleation or initiation) as well as increased myosin activity and intracellular pressure (bleb expansion) and is reversed by subsequent reformation of an actin cortex at the blebbed membrane and myosin-driven retraction. While generally considered as a hallmark of apoptosis, blebbing is also involved in cell migration and cytokinesis (Charras, 2008; Paluch and Raz, 2013). The molecular details of these events are little understood but activation of the small G protein Rho by extra- or intracellular signals, its subsequent activation of the effector kinase Rho-associated kinase (ROCK), and phosphorylation of myosin light chain by ROCK appear to be central processes. In addition, proteins and lipids (such as PIP2) influencing the cortex-membrane interaction and alterations in the cell adhesion properties appear to play a role (Fackler and Grosse, 2008). P2X7 receptor activation causes reversible blebbing in native and recombinant systems (MacKenzie et al., 2001; Mackenzie et al., 2005). This effect is dependent on the P2X7 tail (Wilson et al., 2002) and in a Y2H screen an interaction with the epithelial membrane protein (EMP)-2 was identified and biochemically confirmed for the related proteins EMP-1, EMP-3, and peripheral myelin protein (PMP)-22, which are all widely expressed (references in Wilson et al., 2002). Overexpression of these proteins in HEK293 cells resulted in an increase of caspase-dependent apoptotic-like behavior and blebbing, although a specific interaction domain or mechanism was not identified. In subsequent studies, RhoA, ROCKI, and p38 MAP kinase (Morelli et al., 2003; Verhoef et al., 2003; Pfeiffer et al., 2004) have been shown to be involved in P2X7-induced blebbing and it was demonstrated that the signaling pathway that leads to blebbing is caspase independent and different from that promoting IL-1β release (Verhoef et al., 2003). However, dependence of blebbing on extracellular Ca^2+^- was inconsistent in different studies and both Ca^2+^-dependent and independent pathways leading to blebbing have therefore been proposed (Mackenzie et al., 2005). According to this model, the faster Ca^2+^-dependent zeiotic form of membrane blebbing is a consequence of local Ca^2+^ overload that via induction of PS-flip leads to the disruption of plasma membrane actin interaction. In favor of this model, deregulation of Ca^2+^ entry as a consequence of CaM binding to the P2X7 C-terminus was found to facilitate blebbing (Roger et al., 2008). How Rho is activated remains unresolved. Based on findings in osteoblasts, it was proposed that P2X7 activation leads via PLD and PLA2 activation to LPA, and LPA, by activation of the LPA receptor (a GPCR), activates Rho (Panupinthu et al., 2007). Involvement of PLA2 activation in addition to an undefined Cl^–^ channel in blebbing is supported by a recent study on macrophages (Janks et al., 2019).

Additional effects of P2X7 on cellular membrane trafficking and organization have been reported. These include microvesiculation (MacKenzie et al., 2001; Bianco et al., 2005; Pizzirani et al., 2007), exosome release (Qu et al., 2009; Barberà-Cremades et al., 2017), phagosome maturation (Fairbairn et al., 2001), and formation of multinucleated giant cells (Lemaire et al., 2006). For review see Qu and Dubyak (2009).

### Kinase Activation

#### Protein kinase C (PKC)

PKCs are a family of serine/threonine kinases and represent central mediators of cytoplasmic signaling cascades that regulate a variety of cellular functions.

Three PKC subfamilies (classical, novel, and atypical) have been determined: The classical (PKCα, PKCβ, PKCγ) and the novel (PKCδ, PKCε, PKCθ, and PKCη) PKCs both require the second messenger DAG for activation. The cPKCs require Ca^2+^ as a cofactor while the nPKCs are Ca^2+^-independent. The aPKCs are independent of Ca^2+^ and DAG. Most PKCs are ubiquitously expressed. Their activation is associated with translocation of the enzyme from the cytosolic fraction to the plasma membrane or cell organelles. Besides mediating signal transduction from the plasma membrane, PKCs have been identified within the nucleus, where their role is less well studied (Lim et al., 2015).

P2X7-dependent translocation of Ca^2+^-dependent PKCs has been described in osteoclasts (Armstrong et al., 2009) and Ca^2+^-independent PKCs have also been involved in P2X7 signaling (Bradford and Soltoff, 2002; Gendron et al., 2003) although the molecular interactions and the source of DAG remained unclear in these studies. P2X7 modulation by PKC and its physical interaction with PKCγ was also suggested (Hung et al., 2005).

#### Mitogen Activated Protein Kinases (MAPK)

MAPKs are serine/threonine-specific protein kinases that are activated by phosphorylation as a result of a multi-level signaling cascade. Three types of MAPKs have been found to be phosphorylated upon P2X7 activation, the closely related extracellular signal regulated kinases ERK1 and ERK2, the c-Jun N-terminal kinases (JNKs), and the p38 MAPK (Hu et al., 1998; Armstrong et al., 2002). The ERK1/2 pathway is best investigated and starts with an extracellular ligand binding to its receptor, which then couples to and activates the small GTPase Ras, which via RAF kinases and mitogen-activated protein kinase kinases (MEK1/2) activates ERK1/2. ERK1/2 can regulate RNA translation and several transcription factors and plays an important role in cell division and proliferation. Activation of P38 and JNK MAPKs is more complex and includes numerous kinases that are mostly shared between both MAPKs. Activating stimuli include inflammatory signals and stress and these kinases are involved in apoptosis, proliferation, and inflammation.

Many studies have shown phosphorylation of these kinases following P2X7 activation (Humphreys et al., 2000; Panenka et al., 2001; Gendron et al., 2003). Activation of ERK1 was suggested to be mediated via Ca^2+^, PI3K, c-Src (Gendron et al., 2003; Auger et al., 2005), and EGF receptor transactivation (Stefano et al., 2007). ERK activation was shown to depend mainly on the N-terminus of P2X7 since it was affected by N- but not C-terminal truncations (Amstrup and Novak, 2003).

#### Cellular Sarcoma Tyrosine Kinase (c-Src)

c-Src is a member of the Src kinase family and a protooncogene. It is via myristoylation associated with the plasma membrane and contains src homology (SH) domains 1–4. Its activation causes dephosphorylation of a tyrosine residue and opening of the SH2, SH3, and kinase domains and autophosphorylation. It can be activated by several membrane proteins and can activate various proteins, including focal adhesion proteins, adaptor proteins, and transcription factors and thereby directly or indirectly activates numerous signaling molecules including MAPKs.

Besides activating downstream signaling pathways, the P2X7 receptor itself could serve as a substrate for kinases or phosphatases. For example, Y343 in TM2 was assumed to be dephosphorylated upon receptor activation, since phenylalanine-substitution of Y343 in rat P2X7-expressing HEK293 cells prevented run-down of agonist-evoked currents as well as the effect of phosphatase inhibitors on currents and onset of membrane blebbing (Kim et al., 2001). Additionally, tyrosine residues Y382, Y383, and Y384 in rat P2X7 were found to be phosphorylated by c-Src (Leduc-Pessah et al., 2017).

#### Phosphoinositide 3-Kinase (PI3Ks)/Protein Kinase B (PKB Also Known as Akt)

PI3Ks are a family of kinases that, upon activation by receptors phosphorylate the hydroxyl group in position 3 of phosphatidylinositol, thereby generating various phosphoinos- itides that are able to recruit signaling proteins with phosphoinositide binding PH domains to membranes. Thus, the PH domains of the serine/threonine kinase protein kinase B and the phosphoinositide-dependent kinase (PDK1) bind to PtdIns(3,4,5)P3 (PIP3) and PtdIns(3,4)P2 (PIP2) and thereby localize to the plasma membrane where they interact.

PKB is a serine/threonine kinase that contains a PH domain, which binds with high affinity to phosphatidylinositol (3,4,5)-trisphosphate (PIP3) and is activated by the phosphoinositide-dependent kinase (PDK) 1 and the mammalian target of rapamycin complex 2 (mTOR2). This results in the activation of multiple substrates including mTOR. PKB is involved in antiapoptotic pathways, glucose metabolism, protein synthesis, and cell proliferation and tightly regulated. Numerous and complex effects of P2X7 on Akt have been reported. For example, in neuroblastoma cells and astrocytes, stimulation of P2X7 lead to Akt activation (Jacques-Silva et al., 2004; Amoroso et al., 2015). In another study on neuroblastoma cells, P2X7 inhibition was associated with neuritogenesis and increased Akt phosphorylation (Gomez-Villafuertes et al., 2009) and in pancreatic cancer cells, P2X7 activation was involved in activation of protein and lipid phosphatases that lead to nuclear Akt depletion and inhibited proliferation (Mistafa et al., 2010). Extra- and intracellular calcium, a c-Src-related tyrosine kinase, PI3K, and CaMKII have been involved in Akt activation by P2X7 (Jacques-Silva et al., 2004; Gomez-Villafuertes et al., 2009).

### Neurotransmitter Release

A wealth of literature describes P2X7 localization and function in neuronal cells and its involvement in the release of various neurotransmitters and gliotransmitters. This effect is generally supposed to be a consequence of P2X7-mediated Ca^2+^ increase and beyond the scope of this review. Excellent overviews are given in Sperlagh and Illes (2014) and Miras-Portugal et al. (2017).

### Role in Gene Transcription

P2X7 has been involved in the activation of several transcription factors, most of which play a role in inflammation.

#### Nuclear Factor κ-Light Chain Enhancer of Activated B Cells (NF-κB)

NF-κB is an ubiquitously expressed protein complex that acts as a rapid primary transcription factor (Zhang et al., 2017) and binds to so-called κB-motifs that are present in numerous regulatory DNA regions. It is activated by stressful and pro-inflammatory stimuli such as cytokines, bacterial and viral antigens via a variety of cell surface receptors and initiates the transcription of genes involved in inflammation, proliferation, or survival. In unstimulated cells, NF-κB is bound in the cytoplasm to the inhibitor of κB (IκB). Phosphorylation of IκB by IκB kinase (IKK) leads to its degradation in the proteasome and enables NF-κB to translocate into the nucleus where it binds to target gene promoter sequences. In addition, activity of NF-κB is modulated by phosphorylation (Christian et al., 2016). Activation of P2X7 has been shown to lead to IκB degradation, NF-κB phosphorylation, nuclear translocation, and induced transcription in NF-κB reporter assays (Ferrari et al., 1997b; Aga et al., 2002; Korcok et al., 2004; Genetos et al., 2011; Kim et al., 2013). The signaling mechanisms have not been conclusively resolved but were suggested to involve ROS generation and caspase activation (Ferrari et al., 1997b), ERK1/2 and Akt, (Tafani et al., 2011), MAP kinases (Aga et al., 2004), and MyD88 (Liu et al., 2011). While it is generally accepted that P2X7-induced caspase 1 activation and subsequent IL-1β maturation requires TLR-induced NF-κB signaling (Kahlenberg et al., 2005), a role for P2X7-induced NF-κB signaling and IL-1β transcription (together with NLRP3 components) has also been shown in sterile inflammation upon mechanical trauma (Albalawi et al., 2017).

#### Nuclear Factor of Activated T Cells (NFAT)

The NFAT family consists of five (NFAT1-NFAT5) members and is related to the REL-NF-κB family of transcription factors (Serfling et al., 2012). NFAT1-NFAT4 are activated via CaM and the phosphatase calcineurin, by cell surface receptors that couple to Ca^2+^ mobilization. NFAT5 is activated by osmotic stress and not further discussed here. In its inactivated state, NFAT is phosphorylated and upon dephosphorylation by calcineurin, translocates to the nucleus. Here, it cooperates with other transcription factors (including the AP-1 and Rel family) to regulate immune function and inflammation as well as cell proliferation, cell differentiation and cancer growth. NFAT phosphorylation and inactivation is regulated by multiple kinases in the nucleus (e.g., GSK3) and/or cytoplasm (e.g., CK1). In addition, several other mechanisms, such as caspase 3 cleavage, can regulate NFAT (Müller and Rao, 2010).

In T cells, where NFAT function is best investigated, ATP release and autocrine or paracrine feedback signaling via P2X7 activation has been shown to lead to NFAT induction and release of IL-2 (Yip et al., 2009). In stimulated B cells, however, NFAT internalization in the nucleus was decreased by P2X7-induced membrane depolarisation (Pippel et al., 2015). In microglia cell lines, P2X7 signaling has been shown to activate NFAT proteins (Ferrari et al., 1999) and more recently, the inflammatory CC-motif chemokine ligand 3 (CCL3) was found to be released as a result of this signaling pathway (Kataoka et al., 2009).

Adinolfi et al. (2009) observed an induction in the expression of NFATc1 by heterologous expression of P2X7 in HEK293 cells that lead to promotion of growth and prevention of apoptosis. This effect was confirmed by P2X7 transfection in cancer cell lines and caused an increase in the tumor size and growth rate (Adinolfi et al., 2012). Likewise, transfection of P2X7 into osteosarcoma cells led to an increase in NFATc1 translocation to the nucleus and its activation has been associated with cell growth and proliferation (Giuliani et al., 2014).

#### Hypoxia Inducible Factor (HIF)

Hypoxia inducible factor is a heterodimeric (HIF-α and HIF-β) transcription factor that is upregulated under conditions of low oxygen availability and is implicated in tumor growth. P2X7-mediated upregulation of HIF-1α and ischemic tolerance was reported after ischemic insult in astrocytes (Hirayama et al., 2015; Hirayama and Koizumi, 2017) and P2X7 downmodulation reduced HIF-1α (Amoroso et al., 2015). HIF-1α has also been proposed to regulate the expression of P2X7 in the hypoxic microenvironment, which via Akt and Erk phosphorylation promotes nuclear translocation of NF-κB and tumor cell invasion (Tafani et al., 2011).

#### Other Transcription Factors

Other transcription factors that were reported to be induced upon P2X7 activation include activator protein 1 (AP-1) (Gavala et al., 2010), the early growth response transcription factors (Egr) (Stefano et al., 2007; Friedle et al., 2011), Runt related factor-2 (Runx2) (Yang et al., 2018), and cyclic AMP response element binding protein (CREB) (Ortega et al., 2011). For additional information please refer to Lenertz et al. (2011).

### Cell Death

P2X7 is involved in different forms of cell death. While it is generally reported to cause apoptosis and/or necrosis, multiple alternative cytotoxic routes like pyroptosis and autophagy have also been described (Dubyak, 2012; Yang et al., 2015; Young et al., 2015). Whereas cell swelling and cytolysis could be explained by the plasma membrane permeabilizing properties of the P2X7 receptor (Surprenant et al., 1996) the exact mechanisms of different other forms of necrosis or apoptosis are not known in detail (for a recent review see Di Virgilio et al., 2017). Nevertheless, typical markers of apoptosis such as cytochrome c release, PS-flip, blebbing, cleavage of caspase-3, caspase-8, and caspase-9 have been observed in various systems (Ferrari et al., 1997a; Humphreys et al., 2000; Mackenzie et al., 2005).

## Direct Protein P2X7 Interactions or Interactions Within Protein Complexes

**FIGURE 2:**
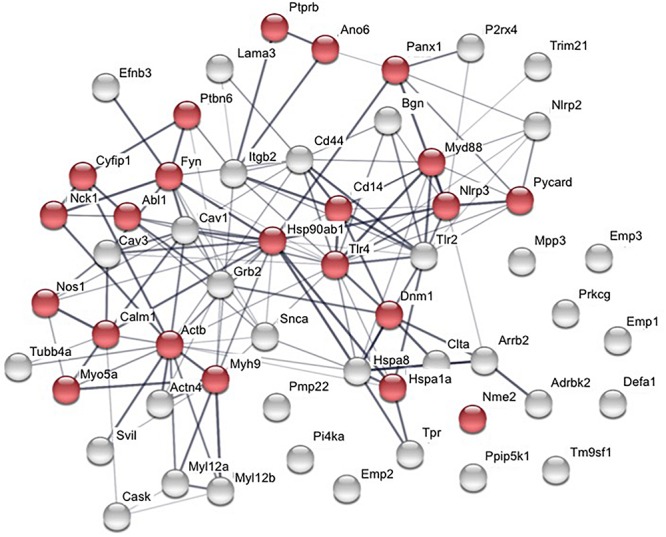
Interaction network of published P2X7 interaction partners. Proteins were analyzed using STRING database (v11.0; Szklarczyk et al., 2019). Twenty-two proteins are involved in function of the innate immune system (shown in red). The thickness of the connecting lines indicates the strength of data support for physical and/or functional associations.

More than 50 proteins have been identified to physically interact with the P2X7 receptor (Table 1). Analysis of their STRING interaction network (Figure 2, Szklarczyk et al., 2019) shows that 22 of these proteins are involved in the innate immune response, in agreement with the proposed pro-inflammatory functions of P2X7. For the majority of the identified proteins, the interaction domains and the physiological consequences of this interactions have not been described. Only interaction partners that were studied in more detail and selected proteins will be briefly described in the following sections.

**TABLE 1:** Published P2X7 interaction partners (adapted from http://www.p2x7.co.uk).

**Gene**	**Protein name**	**Uniprot ID (human)**	**Method**	**Cell system**	**References**
ABL1	Tyrosine-protein kinase ABL1	P00519	Peptide array	*In vitro*	Wu et al., 2007
ACTB^∗^	Actin, cytoplasmic 1 (β-actin)	P60709	IP-MS/WB	HEK293	Kim et al., 2001
			IP-MS	THP-1	Gu et al., 2009
ACTN4^∗^	α-actinin 4	O43707	IP-MS/WB	HEK293	Kim et al., 2001
ANO6	Anoctamin-6	Q4KMQ2	IP-WB	HEK293	Ousingsawat et al., 2015
ARRB2	β-arrestin 2	P32121	IP-WB	CaSKI / HEK293	Feng et al., 2005
Bgn	Biglycan	P21810	IP-WB	Peritoneal macrophages	Babelova et al., 2009
CALM1^∗^	Calmodulin	P0DP23	IP-WB	HEK293	Roger et al., 2008
					Roger et al., 2010
CASK	Peripheral plasma membrane protein CASK	O14936	Y2H	Liver cDNA library	Wang et al., 2011
Cav1/3	Caveolin-1	Q03135	PD/IP-WB	Alveolar epithelial E10 cells	Barth et al., 2008
			nPAGE/IP-WB	Alveolar epithelial E10 cells	Weinhold et al., 2010
			nPAGE-WB	HL-1	Pfleger et al., 2012
	Caveolin-3	P56539	nPAGE-WB	HL-1	Pfleger et al., 2012
CD14	Monocyte differentiation antigen CD14	P08571	IP-WB	HEK293	Dagvadorj et al., 2015
CD44	CD44 antigen	P16070	IP-WB	CHO-K1	Moura et al., 2015
CLTA/B/C/D	Clathrin		IP-WB	CaSKI / HEK293	Feng et al., 2005
CYFIP1	Cytoplasmic FMR1-interacting protein 1	Q7L576	IP-WB	Mouse prefrontal cortex	Li et al., 2017
DEFA1	Neutrophil defensin 1	P59665	PD-WB	HEK293	Chen et al., 2014
DNM1	Dynamin-1	Q05193	IP-WB	CaSKI / HEK293	Feng et al., 2005
EFNB3	Ephrin-B3	Q15768	Y2H	Liver cDNA library	Wang et al., 2011
EMP1/2/3	Epithelial membrane protein 1/2/3	P54849, P54851, P54852	Y2H, PD/IP-WB	HEK293	Wilson et al., 2002
Fyn	Tyrosine-protein kinase Fyn	P06241	IP-WB	OPCs, HEK293	Feng et al., 2015
GRB2	Growth factor receptor-bound protein 2	P62993	Peptide array	*In vitro*	Wu et al., 2007
GRK3	β-adrenergic receptor kinase 2	P35626	IP-WB	CaSKI / HEK293	Feng et al., 2005
HSP90AB1^∗^	Heat shock protein HSP 90-β	P08238	IP-MS/WB	HEK293	Kim et al., 2001
			IP-WB	HEK293, peritoneal macrophages	Adinolfi et al., 2003
			IP-MS	HEK293	Gu et al., 2009
			IP-WB	PC12	Franco et al., 2013
HSPA1A^∗^	Heat shock 70 kDa protein 1A/1B	P0DMV8	IP-MS/WB	HEK293	Kim et al., 2001
			IP-MS	HEK293	Gu et al., 2009
HSPA8^∗^	Heat shock cognate 71 kDa protein	P11142	IP-MS/WB	HEK293	Kim et al., 2001
ITGB2	Integrin β-2	P05107	IP-MS/WB	HEK293	Kim et al., 2001
LAMA3	Laminin subunit α-3	Q16787	IP-MS/WB	HEK293	Kim et al., 2001
MPP3	MAGUK p55 subfamily member 3	Q13368	IP-MS/WB	HEK293	Kim et al., 2001
MYH9^∗^	Myosin-9 (Myosin heavy chain, non-muscle IIa)	P35579	IP-MS/WB	THP-1	Gu et al., 2009
MyD88	Myeloid differentiation primary response protein MyD88	Q99836	IP-WB	HEK293	Liu et al., 2011
MYL12A/B^∗^	Myosin regulatory light chain 12A, Myosin regulatory light chain 12B	P19105 O14950	IP-MS	THP-1	Gu et al., 2009
MYO5A	Unconventional myosin-Va	Q9Y4I1	IP-MS/WB	HEK293	Gu et al., 2009
NCK1	Cytoplasmic protein NCK1	P16333	Peptide array	*In vitro*	Wu et al., 2007
NLRP2/3	NACHT, LRR and PYD domains-containing protein 2	Q9NX02	IP-WB	Astrocytes	Minkiewicz et al., 2013
	NACHT, LRR and PYD domains-containing protein 3	Q9NX02	IP-WB	N13 microglia	Franceschini et al., 2015
NME2	Nucleoside diphosphate kinase B	P22392	IP-MS	HEK293	Gu et al., 2009
NOS1	Nitric oxide synthase, brain	P29475	IP-WB	Mouse brain	Pereira et al., 2013
P2RX4	P2X4 Receptor	Q99571	IP-WB	HEK293, BMDM	Guo et al., 2007
			IP-WB	BMDM	Boumechache et al., 2009
			nPAGE/IP-WB	Alveolar epithelial E10 cells	Weinhold et al., 2010
			PD/IP-WB	tsA 201	Antonio et al., 2011
			IP-WB	Primary gingival epithelial cells	Hung et al., 2013
			IP-WB	HEK293	Pérez-Flores et al., 2015
PANX1	Pannexin-1	Q96RD7	IP-WB	HEK293	Pelegrin and Surprenant, 2006
			IP-WB	J774.2	Iglesias et al., 2008
			IP-WB	Primary neurons	Silverman et al., 2009
			IP-WB	HEK293	Li et al., 2011
			IP-WB	N2a	Poornima et al., 2012
			IP-WB	HPDL	Kanjanamekanant et al., 2014
			PD-WB	N2a	Boyce and Swayne, 2017
PI4KA	Phosphatidylinositol 4-kinase α	P42356	IP-MS/WB	HEK293	Kim et al., 2001
PPIP5K1	Inositol hexakisphosphate and diphosphoinositol-pentakisphosphate kinase 1	Q6PFW1	IP-MS	THP-1	Gu et al., 2009
PMP22	Peripheral myelin protein 22	Q01453	Y2H, PD-WB	HEK293	Wilson et al., 2002
PRKCG	Protein kinase C γ type	P05129	IP-WB	Astrocyte cell line (RBA-2)	Hung et al., 2005
PTPN6	Tyrosine-protein phosphatase non-receptor type 6	P29350	IP-MS	THP-1	Gu et al., 2009
PTPRB	Receptor-type tyrosine-protein phosphatase β	P23467	IP-MS	HEK293	Kim et al., 2001
PYCARD	Apoptosis-associated speck-like protein containing a CARD (ASC)	Q9ULZ3	IP-WB	Primary neurons	Silverman et al., 2009
			IP-WB	Astrocytes	Minkiewicz et al., 2013
Snca	α-synuclein	P37840	IP-WB	Microglia cell line BV2	Jiang et al., 2015
SVIL	Supervillin	O95425	IP-MS/WB	HEK293	Kim et al., 2001
Tlr2/4	Toll-like receptor 2/4	O60603 O00206	IP-WB	Peritoneal macrophages	Babelova et al., 2009
TM9SF1	Transmembrane 9 superfamily member 1	O15321	Y2H	Liver cDNA library	Wang et al., 2011
TPR	Nucleoprotein TPR	P12270	IP-MS	HEK293	Gu et al., 2009
TRIM21^∗^	E3 ubiquitin-protein ligase TRIM21 (52 kDa Ro protein)	P19474	IP-MS	THP-1; HEK293	Gu et al., 2009
TUBB^∗^	Tubulin β chain	P07437	IP-MS	HEK293	Gu et al., 2009

*Asterisks indicate proteins that are frequently found as contaminants in MS-based approaches (Mellacheruvu et al., 2013). PD, pull down; IP, immunoprecipitation; WB, western blot; MS, mass spectrometry; nPAGE, native PAGE; Y2H, yeast two-hybrid.*

### Proteins Involved in P2X7-Mediated Interleukin Secretion

Pathogen-associated molecular patterns like LPS activate innate immune responses via binding to TLR4. CD14 serves as a co-receptor of TLR4 to facilitate the cellular responses to LPS (Zanoni and Granucci, 2013). As described [see Section “Trafficking and Lipid Interaction Domains (∼residues 540–595)”], P2X7 harbors a potential LPS binding motif in its C-terminal domain (Denlinger et al., 2001) and CD14 was identified as a potential co-receptor of P2X7 that enables LPS internalization and binding to P2X7. Their physical interaction was shown in immunoprecipitation experiments with transfected HEK293 cells. LPS stimulation increased their co-localization and the amount of co-precipitated CD14 or P2X7 proteins (Dagvadorj et al., 2015).

MyD88 is another protein that is tightly associated with TLR function. TLR4 can signal through MyD88 to induce the synthesis of pro-inflammatory cytokines via the activation of NF-κB. In transfected HEK293 cells, it was shown that MyD88 physically interacts with P2X7, suggesting that MyD88 is responsible for P2X7-mediated NF-κB activation. The C-terminus of P2X7 and, in particular, the amino acid G586 was shown to be important for this interaction. Alanine substitution of G586 lead to a loss of P2X7 function, decreased caspase 1 cleavage activity, altered cellular localization, and impaired interaction between P2X7 and MyD88 in mouse, P2X7-expressing HEK293 cells and RAW264.7 cells (Liu et al., 2011).

Inflammation is not only triggered by the binding of exogenous PAMPs to TLR4, but also mediated via endogenous structures. Soluble biglycan, a proteoglycan of the extracellular matrix, can activate TLR2 and 4 and stimulate inflammatory responses (Schaefer et al., 2005; Moreth et al., 2014). Co-precipitation experiments showed that biglycan can directly interact with P2X4 and P2X7 in peritoneal macrophages (Babelova et al., 2009). Interestingly, also TLR2 and 4 were co-precipitated with anti-P2X4 or anti-P2X7 antibodies in the presence of biglycan.

P2X7 was also found to directly interact with components of the inflammasome such as the adaptor protein ASC of the NLRP1 inflammasome in neurons (Silverman et al., 2009), ASC, and the NLR subunit of the NLRP2 inflammasome in astrocytes (Minkiewicz et al., 2013), and NLRP3 in N13 mouse microglial cells (Franceschini et al., 2015). For NLRP3 and P2X7 also a mutual relationship in mRNA and protein levels was detected and both proteins co-localize at discrete sites in the subplasmalemmal cytoplasm (Franceschini et al., 2015).

### Pannexin-1

Pannexin-1 belongs to the pannexin family of channel-forming glycoproteins and has been reported to mediate the release of ATP (Chekeni et al., 2010). It can be activated by various stimuli (e.g., mechanical, caspase cleavage, cytoplasmic Ca^2^^+^, membrane depolarization, extracellular ATP, and K^+^; Penuela et al., 2013) and has been proposed to form the P2X7-associated macropore (Pelegrin and Surprenant, 2006). Indeed, several co-precipitation experiments revealed a physical interaction with the P2X7 receptor (Pelegrin and Surprenant, 2006; Iglesias et al., 2008; Silverman et al., 2009; Li et al., 2011; Poornima et al., 2012; Kanjanamekanant et al., 2014; Boyce and Swayne, 2017). However, negative pull-down experiments were also reported and more recent studies indicate that the macropore is an intrinsic property of P2X7 and opens immediately upon activation (Harkat et al., 2017; Karasawa et al., 2017; Pippel et al., 2017; Di Virgilio et al., 2018b). A functional interaction of P2X7 and pannexin in inflammasome activation was also described (Pelegrin and Surprenant, 2006; Locovei et al., 2007; Iglesias et al., 2008; Hung et al., 2013; Boyce et al., 2015; Boyce and Swayne, 2017) but is under discussion (Qu et al., 2011; Hanley et al., 2012; Alberto et al., 2013). As pannexin is considered to mediate the release of ATP, it might play a role upstream of P2X7 by controlling its activation (Chekeni et al., 2010; Isakson and Thompson, 2014).

### Heat Shock Protein 90

Heat shock protein (HSP) 90 is a molecular chaperone and ATPase and one of the most abundant cytosolic proteins in eukaryotes. It is essential for protein folding and maturation and has been involved in many different pathologies, including infections, cancer, and neurodegenerative diseases (Schopf et al., 2017). Two independent MS-based screening approaches in HEK293 cells identified HSP90 as potential interactor of P2X7 (Kim et al., 2001; Gu et al., 2009) and the cysteine-rich domain in the C-terminus was identified to be important for this interaction (Migita et al., 2016). Phosphorylation of HSP90 was shown to decrease P2X7 currents and membrane blebbing in HEK293 cells and rat peritoneal macrophages (Adinolfi et al., 2003). Nitration of the chaperone increased P2X7-dependent activation of the Fas pathway and subsequent apoptosis in PC12 cells (Franco et al., 2013). Fas (CD95) is a member of the tumor necrosis factor receptor (TNFR) superfamily and plays a central role in apoptosis. HSP90 was also found to be involved in P2X7 pore formation and P2X7-dependent autophagic death of dystrophic muscles (Young et al., 2015) as well as the activation of the P2X7/NLRP3 inflammasome pathway (Zuo et al., 2018). It was shown that HSP90 directly interacts with the LRR and NACHT domains of NLRP3 and is essential for inflammasome function and activity (Mayor et al., 2007).

### Caveolin

Caveolins are the most abundant membrane proteins in caveolae and act as scaffolding and membrane curvature inducing proteins. Caveolae are invaginations of the plasma membrane and, similar to lipid rafts, enriched in cholesterol and glycosphingolipids (Patel and Insel, 2008). The caveolin family comprises three family members (caveolin-1, -2, -3). In E10 alveolar epithelial cells, P2X7 was found to be associated with caveolae and partially co-localized with caveolin-1 (Barth et al., 2007). A direct interaction of both proteins was shown via co-precipitation (Barth et al., 2008). This interaction was further verified via native PAGE, which indicated that both proteins are present in the same protein complex (Weinhold et al., 2010). Similar results were obtained in cardiomyocytes, where also caveolin-3 was detected (Pfleger et al., 2012). A mutual relation in expression and localization was shown (Barth et al., 2007; Weinhold et al., 2010).

### Anoctamin Channels

Anoctamin channels (TMEM16 family) are calcium-activated Cl^–^ channels and are co-expressed with P2X7 in various cell types. Since a P2X7-mediated increase in anion conductance has been observed in several studies, a physical or functional interaction with anoctamin channels was investigated (Stolz et al., 2015). In *Xenopus laevis* and *Ambystoma mexicanum* oocytes [which lack endogenous anoctamin(s)], a functional interaction between heterologously expressed P2X7 and anoctamin-1 could be shown, but not for anoctamin-6 (Stolz et al., 2015). However, another study in the same year could show a P2X7-mediated activation of anoctamin-6 in *Xenopus laevis* oocytes, transfected HEK293 cells, and mouse macrophages and a physical interaction was also shown in co-immunoprecipitation experiments with transfected HEK293 cells (Ousingsawat et al., 2015).

### Calmodulin

A novel CaM binding motif was identified in the C-terminus of rat P2X7 [compare Section “Trafficking and Lipid Interaction Domains (∼Residues 540–595)”]. The specific binding of CaM to this region was shown by co-immunoprecipitation and mutagenesis of the binding motif in HEK293 cells (Roger et al., 2008). Interestingly, this binding motif is absent in human and mouse P2X7 and indeed, an interaction of CaM with the human receptor could not be detected but reconstituted by mutagenesis (Roger et al., 2010). The binding of CaM facilitates and prolongs Ca^2+^ entry and was proposed to play a role in cytoskeletal rearrangements and membrane blebbing (Roger et al., 2008).

### Myosin-9

The non-muscle myosin-9 was not only shown to co-precipitate with P2X7, but also to co-localize in the plasma membrane and membranes of intracellular organelles in a human monocytic cell line (THP-1 cells). A close association with P2X7 was confirmed by FRET experiments in HEK293 cells (Gu et al., 2009). It was proposed that P2X7 is anchored in the membrane by myosin-9 and activation of P2X7 via extracellular ATP leads to dissociation of the myosin-P2X7 complex and the formation of the large pore and membrane blebbing. It was further suggested that the integrity of this complex is required to regulate P2X7-mediated phagocytosis (Gu et al., 2010).

## Conclusion

Blockade or genetic deletion of the P2X7 receptor has shown positive effects in numerous disease models and genetic association studies have linked SNPs of this receptor with various human diseases. While P2X7-induced cytokine secretion and/or cell death have been identified as important mechanisms that contribute to its pathophysiological role, the relevance and in particular, the molecular mechanism leading to the induction of many other identified P2X7-induced effects remain much less investigated. The extended P2X7 C-terminus has been involved in many P2X7-specific functions and is supposed to constitute a platform for intracellular interactions that initiate multiple signaling pathways. Although more than 50 interacting proteins have been identified (Table 1), their roles in receptor signaling, trafficking, regulation, or modification remain largely obscure and in most cases, the sites of interaction, the aa involved, and the molecular mechanisms are unknown. Many of the identified interactions are likely to depend on the type and/or state of the cell and might also be indirect or due to the association of proteins in larger domains or complexes (e.g., lipid rafts). However, the data need to be interpreted with caution as they include proteins that tend to interact with the solid-phase support or the used affinity tags and are frequently found as contaminants in affinity purification approaches followed by mass spectrometry (MS) (Mellacheruvu et al., 2013) (marked with asterisk in Table 1). It further has to be considered, that several proteins were identified in targeted rather than unbiased screening approaches and most experiments were carried out in heterologous expression systems (in some of which P2X7 is not naturally occurring) and with overexpressed interaction partners, which might bear the risk of artificial aggregation.

In contrast to other receptor complexes, for which interaction partners have been defined (Schwenk et al., 2012, 2016; Hanack et al., 2015), few tight interactions that survived purification were identified for P2X7 and BN-PAGE analysis in mouse and rat tissues did not reveal bands that are reconcilable with complexes containing additional proteins besides the three P2X7 subunits (Nicke, 2008). Of the interaction partners identified in pull-down experiments, only few have been repeatedly identified or confirmed in independent studies. Thus, P2X7 interactions or complexes appear to be rather instable and the P2X7 tail might mainly have a structural role and/or serve as a scaffold for temporary and short-lived interactions in which Ca^2+^ signaling and interactions with membrane components are likely to play a major role. The specific molecular mechanisms involved are largely hypothetical and only few interaction sites have been determined by mutagenesis. Elucidation of these interactions and the downstream signaling pathways involved bears the potential to identify novel ways for therapeutic intervention.

## Author Contributions

AN conceived and supervised the project. All authors wrote, reviewed, and approved the manuscript. RK prepared the table. AK and RK designed the figures.

## Conflict of Interest Statement

The authors declare that the research was conducted in the absence of any commercial or financial relationships that could be construed as a potential conflict of interest.
